# Periconceptional Maternal Protein Intake from Animal and Plant Sources and the Impact on Early and Late Prenatal Growth and Birthweight: The Rotterdam Periconceptional Cohort

**DOI:** 10.3390/nu14245309

**Published:** 2022-12-14

**Authors:** Sofie van Zundert, Simone van der Padt, Sten Willemsen, Melek Rousian, Mina Mirzaian, Ron van Schaik, Régine Steegers-Theunissen, Lenie van Rossem

**Affiliations:** 1 Department of Obstetrics and Gynecology, Erasmus MC, University Medical Center, P.O. Box 2040, 3000 CA Rotterdam, The Netherlands; 2 Department of Clinical Chemistry, Erasmus MC, University Medical Center, P.O. Box 2040, 3000 CA Rotterdam, The Netherlands; 3 Department of Biostatistics, Erasmus MC, University Medical Center, P.O. Box 2040, 3000 CA Rotterdam, The Netherlands

**Keywords:** periconception period, pregnancy, nutrition, crown-rump length, embryonic volume, estimated fetal weight, birthweight

## Abstract

Plant-based diets continue to rise in popularity, including among women of reproductive age, while consequences for pregnancy outcomes have hardly been studied. During pregnancy, maternal diet is the only source of proteins for the developing fetus. Hence, we investigated the effects of periconceptional maternal animal and plant protein intake on prenatal growth and birthweight. 501 pregnancies were included from the prospective Rotterdam Periconceptional Cohort. Embryonic growth was depicted by crown-rump length (CRL) and embryonic volume (EV) at 7, 9 and 11 weeks using 3D ultrasound scans. Estimated fetal weight (EFW) at 20 weeks and birthweight were retrieved from medical records and standardized. Multivariable mixed models were used for CRL and EV trajectories, and linear regression for EFW and birthweight. A 10 g/day higher maternal animal protein intake was positively associated with increased embryonic growth (CRL: *β* = 0.023 √mm, *p* = 0.052; EV: *β* = 0.015 ∛cm, *p* = 0.012). A positive association, albeit non-significant, was found between maternal animal protein intake and EFW, and birthweight. No clear associations emerged between maternal plant protein intake and prenatal growth and birthweight, with effect estimates close to zero. In conclusion, maternal animal protein intake during the periconception period was positively associated with early and late prenatal growth and birthweight, while no associations were found between maternal plant protein intake and prenatal growth and birthweight.

## 1. Introduction

In recent years, there has been a shift towards diets consisting of fewer animal products and more plant-based foods due to their lower impact on environment and climate change [1]. It is widely known that the protein quality, which is determined by its ability to meet the requirements for essential amino acids and its digestibility, of plant proteins is generally lower than that of animal proteins [2]. However, adherence to plant-based diets reduces the incidence of non-communicable diseases in the general population. So far, less attention has been devoted to the potential risks of adherence to plant-based diets during pregnancy [3,4,5]. Maternal dietary quality is especially important during the periconception period, which is defined as the 14 weeks before conception until the 10 weeks thereafter. During this critical time window, epigenetic programming can affect the development of the gametes, embryo and placenta, and can induce long-term health effects on the offspring [6].

Our earlier studies have shown that periconceptional maternal dietary patterns have an impact on reproduction and prenatal growth and development [7]. Existing research on maternal protein intake has mainly focused on birthweight and most studies revealed a positive association [8,9,10,11]. In contrast, Morisaki, et al. [12] found a non-linear (inverse U-curve) association between maternal protein intake and fetal growth and birthweight. A few studies have distinguished between animal and plant proteins [8,10,11]. A higher birthweight and a lower risk of fetal growth restriction was found in women who consumed more animal proteins during pregnancy. The associations between plant proteins and fetal growth and birthweight were, however, less evident and inconsistent [8,10,11]. Despite the importance of embryonic growth as a determinant for fetal growth, birthweight and health later in life, the relationship between periconceptional maternal protein intake and embryonic growth is unknown [13,14,15].

Since maternal diet is the primary source of proteins required for embryonic and fetal growth, we hypothesize that periconceptional maternal protein intake is positively associated with prenatal growth and birthweight. Considering the higher protein quality of animal proteins compared to plant proteins, we hypothesize that the associations are stronger for animal proteins than for plant proteins when distinguishing between protein source. The objectives of this study are to determine the associations between periconceptional maternal protein intake, including animal and plant protein intake, and embryonic growth. A secondary objective is to investigate the relationship with fetal growth and birthweight.

## 2. Materials and Methods

### 2.1. Study Design and Participants

The data used for this study were collected prospectively within the Rotterdam Periconceptional Cohort (Predict Study), an ongoing periconceptional prospective tertiary hospital-based cohort study conducted at the Department of Obstetrics and Gynecology of the Erasmus MC, University Medical Center, Rotterdam, The Netherlands (Erasmus MC) from 2010 onwards. Approval for the Predict Study was granted by the Central Committee on Research in The Hague and the local Medical Ethics Committee of the Erasmus MC (15 October 2004, MEC-2004-277). All participants gave written informed consent before inclusion [7,16]. Data on periconceptional maternal protein intake from animal and plant sources have been obtained from the Food Frequency Questionnaire (FFQ) since November 2014 [17]. Therefore, only women who entered the Predict Study from November 2014 onwards were included in this study. Additionally, women had to be 18 years or older, familiar with the Dutch language and less than 8 weeks pregnant.

**Figure 1:**
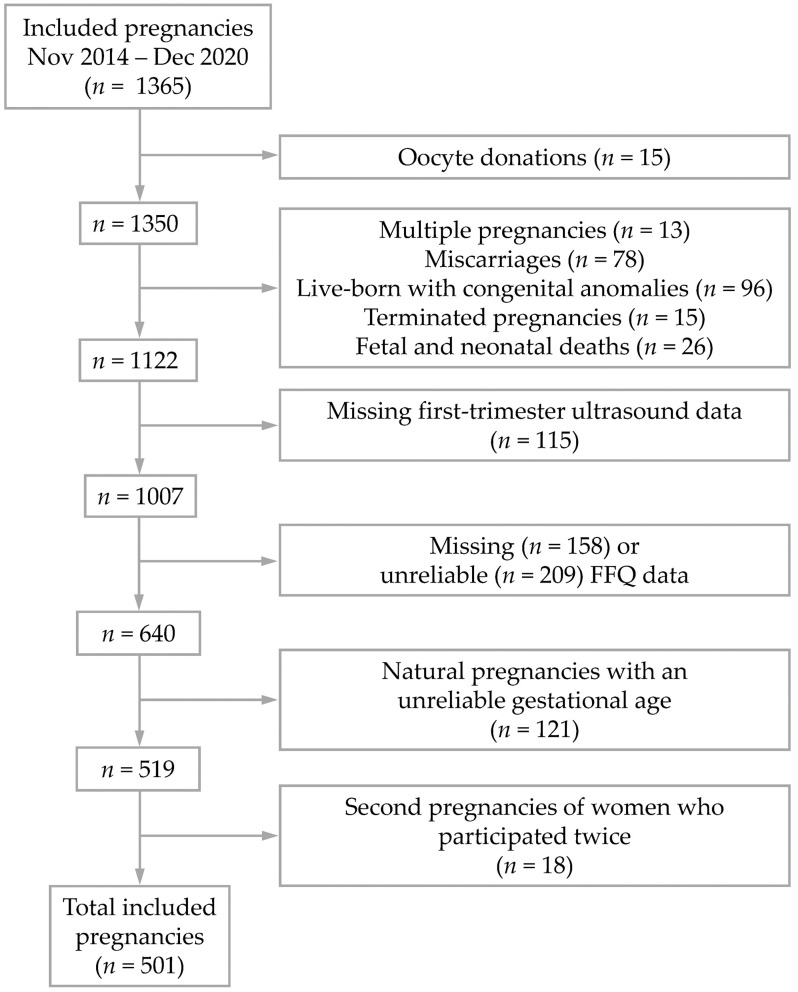
Selection of the study population from the Rotterdam Periconceptional cohort (Predict Study). FFQ = Food Frequency Questionnaire.

In total, 1365 pregnancies were included between November 2014 and December 2020. Pregnancies conceived after oocyte donation (*n* = 15) were excluded, because no information on maternal diet of the donors was collected. Furthermore, multiple pregnancies (*n* = 13) and pregnancies with an a-priori increased risk of impaired embryonic growth were excluded: miscarriages (*n* = 78), live-born with congenital anomalies (*n* = 96), terminated pregnancies because of congenital anomalies (*n* = 15), and fetal and neonatal deaths (*n* = 26) [18,19]. Pregnancies were also excluded when missing first-trimester ultrasound data (*n* = 115), missing FFQ data (*n* = 158) or unreliable FFQ data (*n* = 209) according to the Goldberg cut-off method explained further in Section 2.2.3. We additionally excluded naturally conceived pregnancies with an unreliable gestational age (GA) (*n* = 121), as GA is an important determinant of embryonic and fetal growth. Our definition of an unreliable GA can be found in Section 2.2.1. When women participated twice within the Predict study, only the first pregnancy (*n* = 18) was included in the current study to avoid clustering. This resulted in a total study population of 501 pregnancies (Figure 1).

### 2.2. Data Collection

#### 2.2.1. Embryonic Growth and Gestational Age

Embryonic growth was depicted by longitudinal CRL and EV measurements using three dimensional (3D) ultrasound scans and virtual reality (VR) techniques performed in the 7th, 9th and 11th week of gestation by trained medical doctors. A comprehensive description of the 3D ultrasound scans and measurements of CRL and EV using VR techniques has been described extensively in previous publications [7,16,20]. The acquired 3D ultrasound scans were transferred and visualized using VR systems by trained researchers following a standard protocol. An interactive 3D hologram is created by these VR systems, allowing real depth perception and precise measurements of the embryo [20]. The CRL measurement is performed manually, while the EV measurement is performed using a semi-automated method. Both CRL and EV are reliable and reproducible measurements, proven by inter-observer and intra-observer variability analyses (intra-class correlation coefficients (ICC) > 0.99) [7,20].

As CRL was a primary outcome measure, it was not used to estimate the GA of the embryo. Naturally conceived pregnancies also included pregnancies after ovulation induction (OI) and intrauterine insemination (IUI). For naturally conceived pregnancies, the GA was calculated from the first day of the last menstrual period (LMP) of a regular menstrual cycle. If the menstrual cycle was <25 days or >31 days, the GA was adjusted for the length of the menstrual cycle. The GA of a naturally conceived pregnancy was considered unreliable when the menstrual cycle was irregular, the LMP was missing or when the calculated GA differed more than 7 days from the estimated GA based on the CRL. For pregnancies conceived after in vitro fertilization (IVF) or intracytoplasmic sperm injection (ICSI), the GA was calculated from the conception date, which was the oocyte retrieval day minus 14 days for fresh embryo transfers, and the embryo transfer day minus 19 days for cryopreserved embryo transfers.

#### 2.2.2. Fetal Growth and Birthweight

Data on fetal growth parameters were retrieved from the routine transabdominal mid-pregnancy anomaly scans at around 20 weeks of pregnancy (*n* = 422). Estimated fetal weight (EFW) was calculated using the Hadlock formula based on the head circumference (HC), abdominal circumference (AC) and femur length (FL) in centimeters [21]:

EFW = 10^1.3596 − 0.00386 × AC × FL + 0.0064 × HC + 0.00061 × BPD × AC + 0.0424 × AC + 0.174 × FL^ (1)

Birthweights were retrieved from medical records. For EFW and birthweight standard scores (*z*-scores) were calculated based on Dutch reference growth curves adjusted for GA, and additionally for fetal sex, respectively [22,23].

#### 2.2.3. Food Frequency Questionnaire

To assess information on periconceptional maternal protein intake, participants received a standardized semi-quantitative FFQ at enrollment. The FFQ was developed at the Division of Human Nutrition and Health, Wageningen University, The Netherlands, and covered the dietary intake of participants during the previous four weeks [24]. The FFQ included questions regarding preparation methods, portion sizes and additions, and was validated for the intake of macronutrients and energy in women of reproductive age [25]. The amount of energy and the nutritional values of the food items were determined using the Dutch food composition table and were presented in commonly used units using Dutch household measures [26]. FFQ data were considered unreliable when the reported energy intake was below the calculated individual’s energy intake based on basal metabolic rate and physical activity (Goldberg cut-off) as proposed by Black [27]. The 95% lower confidence limit of the calculated Goldberg cut-off provided the cut-off energy intake for each individual. A more detailed description of the rationale and calculation of the Goldberg cut-off can be found in the recently published study of Smit et al. [28].

#### 2.2.4. Maternal Characteristics

Data on maternal characteristics such as date of birth, geographical background, educational level, parity, conception mode and periconceptional lifestyle factors (smoking, alcohol consumption, drug use and folic acid supplement use) were obtained from general questionnaires filled out by participants before their intake appointment in the first trimester of pregnancy. Height and weight were measured by a research nurse at the intake appointment in the first trimester of pregnancy, and body mass index (BMI) was calculated by dividing weight (kg) by the square of height (m). Age at conception was calculated by the difference between the date of birth and the conception date as described in Section 2.2.1. Geographical background was categorized into western and non-western and educational level as high, intermediate or low according to the Dutch central bureau of statistics (CBS) [29,30]. Parity was divided into nulliparous and multiparous. Conception mode was verified by medical records and divided into naturally conceived pregnancies and IVF/ICSI pregnancies. Folic acid supplement use was considered adequate when started before conception.

### 2.3. Statistical Analysis

The baseline characteristics were presented as means with standard deviation (SD) or as numbers of individuals with percentages. To study differences in baseline characteristics between those with a low and those with a high protein intake relative to their energy intake, participants were divided into tertiles based on protein intake as percentage of energy intake.

To illustrate whether the protein intake of our study population met the recommended daily intake (RDI), the RDI was calculated for each individual per trimester of pregnancy according to the EFSA Panel on Dietetic Products [31]:

RDI (g/day) = 0.83 × weight (kg) *                 * +1 g/day in the first trimester, +9 g/day in the second trimester, and +28 g/day in the third trimester of pregnancy           (2)

Model 1 included GA and energy intake, and animal and plant protein intake were mutually adjusted for each other. Model 2 (the adjusted model) was additionally adjusted for the following covariates based on previous research and a Directed Acyclic Graph: age, BMI, geographical background, educational level, parity, conception mode, fetal sex, smoking, and folic acid supplement use [32,33,34,35,36,37,38,39]. The percentage of missing values of the covariates did not exceed 5%. Since GA is the strongest determinant of prenatal growth, it was further evaluated by adding a cubic spline function for GA and interaction terms between GA and all the other above mentioned covariates. Only the non-linear GA improved our model fit and was included in all models.

Mixed models were used to determine the associations between periconceptional maternal protein intake and longitudinal CRL and EV measurements, taking into account subject-specific trajectories. CRL and EV were transformed (√CRL and ∛EV) to obtain a constant variance and a normal distribution of the residuals given the covariates. Three different types of sensitivity analyses were performed to assess the robustness of the main findings. First, the nutrient density method was used to correct for differences in protein intake due to differences in energy intake [40]. Model 1 and 2 were again used for analyses, but included protein intake as percentage of energy intake instead of grams per day. Next, as animal protein sources contain generally more iron than plant protein sources, which is an important micronutrient for prenatal growth, iron intake could potentially explain the effect of animal protein intake [5]. Therefore, as a second sensitivity analysis iron intake was additionally added to Model 2 (Model 3). Finally, in the last sensitivity analysis, analyses using Model 1 and Model 2 were repeated only in women with a BMI within the normal range (18.5–24.9 kg/m^2^), who are least likely to under- or over-report their dietary intake [41,42].

Linear regression models were used to determine the associations between periconceptional maternal protein intake with EFW and birthweight. For EFW and birthweight, standard scores (*z*-scores) were calculated based on Dutch reference growth curves [22,23]. The reference curve of EFW was adjusted for GA and that of birthweight for GA and fetal sex. Except for the covariates GA and fetal sex, the models were built up in the same way as Model 1 and Model 2 with embryonic growth as outcome. In short, Model 1 included energy intake, and either maternal animal or plant protein intake (mutually adjusted for each other). Model 2 also included the covariates age, BMI, geographical background, educational level, parity, conception mode, fetal sex (only in models with standardized EFW as outcome), smoking, and folic acid supplement use.

All analyses were performed using R version 4.2.1. of R Core Team [43]. Results were reported as effect estimates with 95% confidence intervals (95% CI). A *p*-value ≤ 0.05 was considered statistically significant.

## 3. Results

### 3.1. Baseline Characteristics

Table 1 shows the baseline characteristics of the total study population and stratified by tertiles of energy intake derived from total protein intake. Women who consumed more total protein as percentage of energy intake were slightly older (T3 vs. T1: 33.3 (3.9) vs. 32.1 (4.5) years) and less frequently low-educated (T3 vs. T1: 2.5% vs. 12.3%). Furthermore, women with a higher energy intake from total protein had a lower total energy intake (T3 vs. T1: 1740 (383) vs. 2100 (699) kcal/day), were more likely to start folic acid supplement use preconceptionally (T3 vs. T1: 91.8% vs. 85.2%), and were less likely to smoke (T3 vs. T1: 8.8% vs. 17.9%) or use drugs (T3 vs. T1: 0.6% vs. 3.7%). Women in the highest tertile of energy intake from total protein were more often western (T3 vs. T1: 90.6% vs. 87.0%) and nulliparous (T3 vs. T1: 45.0% vs. 39.5%), and conceived after IVF/ICSI (T3 vs. T1: 57.5% vs. 52.7%) compared with those in the lowest tertile. No dose-response relationship was found between total protein intake as percentage of energy intake and BMI (T3 vs. T1: 24.6 (3.5) vs. 24.7 (4.2) kg/m^2^), and alcohol consumption (T3 vs. T1: 25.0% vs. 24.1%).

The baseline characteristics of the included (*n* = 501) and excluded population (*n* = 864) are shown in Table S1 (Supplementary Material). In contrast to the included pregnancies, the majority of the excluded pregnancies were naturally conceived (64.3% vs. 45.9%). Furthermore, excluded women more often had a non-western geographical background (16.6% vs. 10.4%), had a higher BMI (27.1 (5.5) vs. 24.7 (4.1) kg/m^2^) despite a lower reported total energy intake (1590 (678) vs. 1940 (562) kcal/day), and started folic acid supplement use less often in the preconception period (75.3% vs. 87.1%) than the included women.

### 3.2. Protein Intake

The intake of protein in our study population is summarized in Table 2. For protein intake, we refer to maternal protein intake during the periconception period. The mean protein intake was 72.8 g/day, which was 13.5 g/day more than the first trimester RDI, 5.5 g/day more than the second trimester RDI, but 13.5 g/day less than the third trimester RDI [31]. Slightly more than half of the total protein intake was derived from animal sources (56.0%). The contribution of protein intake to energy intake was 15.2%, of which animal protein intake accounted for 8.6% and plant protein intake for 6.5%.

### 3.3. Embryonic Growth

**Figure 2:**
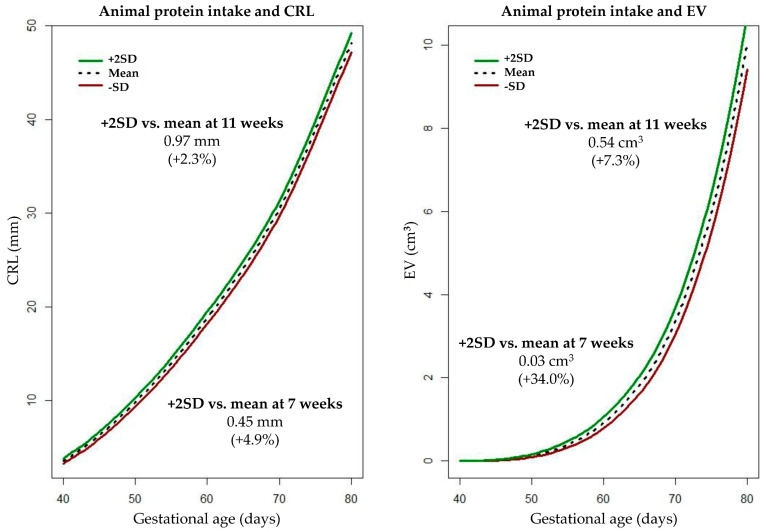
Embryonic growth according to periconceptional maternal animal protein intake. CRL = Crown-Rump Length, EV = Embryonic Volume.

Model 1 shows a positive association between total protein intake and embryonic growth (Table 3). After adjustment for covariates (Model 2), this positive association persisted with comparable effect estimates. These effect estimates indicate that a 10 g/day higher total protein intake increases the √CRL by 0.022 mm (*p* = 0.061) and ∛EV by 0.014 cm^2^ (*p* = 0.17), respectively. Furthermore, animal protein intake was positively associated with embryonic growth in both the unadjusted (Model 1) and adjusted (Model 2) analysis. In the adjusted analysis (Model 2), a 10 g/day higher animal protein intake was associated with an increase of √CRL and ∛EV by 0.023 mm (*p* = 0.052) and 0.015 cm^2^ (*p* = 0.012), respectively. In essence, this means that in the total study population, at 11 weeks of pregnancy, the difference in CRL between −2SD and +2SD protein intake is 2.493 mm, which is an increase of 6.0% in CRL, and corresponds with 0.2 days according to Verburg, et al. [44]. Figure 2 illustrates the positive associations of animal protein intake with CRL and EV trajectories. No statistically significant associations were found between plant protein intake and embryonic growth.

A series of sensitivity analyses were performed. First, the nutrient density method was used to correct for differences in protein intake due to differences in energy intake. Analyses with protein intake as percentage of energy intake increased the effect estimates and decreased the width of the confidence intervals, suggesting more precise estimates (Table 4). Adjustment for covariates did not influence the associations much. The adjusted analysis (Model 2) showed that a 10% higher total protein intake increased √CRL by 0.119 mm (*p* = 0.035) and ∛EV by 0.061 cm^2^ (*p* = 0.024). Similarly, a 10% higher animal protein intake increased √CRL and ∛EV by 0.110 mm (*p* = 0.049) and 0.062 cm^2^ (*p* = 0.021), respectively. In the adjusted model including iron intake (Model 3), the effect estimates were quite similar to those of Model 2, suggesting that iron intake did not affect the relationship (Table 5). Lastly, the effect estimates were slightly stronger in women with a normal BMI (18.5–24.9 kg/m^2^), but were not substantially different from the associations in the total study population, indicating limited bias by under- or over-reporting of dietary intake (Table 6).

### 3.4. Fetal Growth and Birthweight

In both the unadjusted and adjusted analyses, no statistically significant associations were found between protein intake and fetal growth and birthweight (Table 7). Though not statistically significant, protein intake was positively associated with fetal growth (Model 1). Adjustment for covariates did not considerably change the estimated associations (Model 2). In the unadjusted analyses (Model 1), protein intake was also positively associated with birthweight. However, after adjustment for covariates the effect estimates decreased and the width of the confidence intervals increased (Model 2).

## 4. Discussion

### 4.1. Main Findings

**Figure 3:**
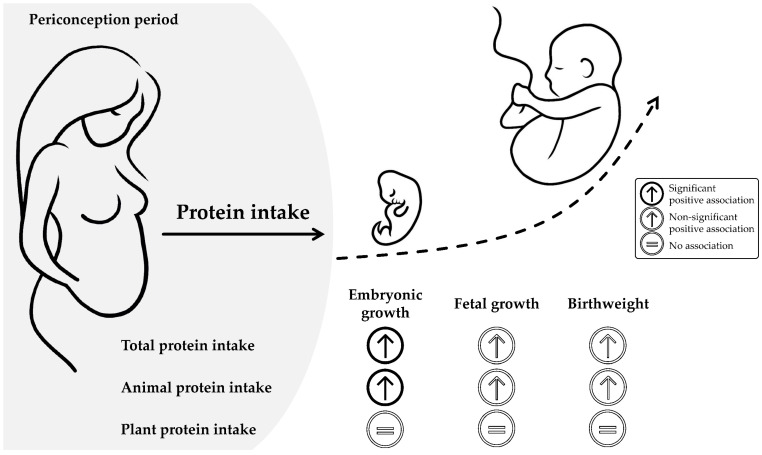
Graphical summary of the findings.

The primary aim of this study was to investigate the associations between periconceptional maternal protein intake from animal and plant sources and embryonic growth. Furthermore, associations with fetal growth and birthweight were examined. This study showed that, even in women with a protein intake that generally met the nutritional requirements, periconceptional protein intake, and in particular from animal sources, was positively associated with embryonic growth. In addition, periconceptional maternal total and animal protein intake were associated with higher EFW and birthweight, though not statistically significantly. In this study, no associations between periconceptional maternal plant protein intake and prenatal growth and birthweight were found (Figure 3).

### 4.2. Interpretation of Findings and Comparison with Previous Studies

#### 4.2.1. Maternal Protein Intake

The positive associations between periconceptional maternal protein intake and embryonic growth, fetal growth and birthweight are in line with previous research, which showed that maternal protein intake was associated with a reduced risk of fetal growth restriction and a higher birthweight [8,9,11,45]. In contrast, one cohort study found a non-linear (inverse U-curve) association between maternal protein intake and birthweight [12]. This inconsistency can be at least partly explained by the study population as it included Japanese women with other dietary habits, and thus protein and energy intake, amino acid composition and micronutrient content were different from our study population, consisting of mainly Dutch women. In the current study, no non-linear function of maternal protein intake was included, since none of the participants had a protein intake below the RDI. It could be that, in a larger more diverse population regarding protein intake, we would have found a non-linear association with prenatal growth and birthweight. The findings of the current study support our hypothesis that periconceptional maternal protein intake plays an important role in prenatal growth. Maternal protein intake is the primary source of amino acids for the developing fetus during pregnancy, which are involved in multiple metabolic pathways. Disruption of these metabolic pathways can cause epigenetic alterations, which may affect the expression of genes involved in growth of the fetus [6,46,47].

Although we did find a positive association between periconceptional maternal protein intake with EFW and birthweight, this association was not statistically significant. The consistency of the direction of the associations with EFW and birthweight suggests a positive trend overall. A possible explanation for attenuation of the effect later in pregnancy might be the transition from histio-trophic nutrition in the first trimester of pregnancy to hemo-trophic nutrition in the second and third trimester of pregnancy [48]. There is increasing evidence that during early pregnancy nutrients for the developing embryo are provided by uterine glands which discharge secretions into the intervillous space, and by accumulation of maternal proteins within coelomic cavity fluid from which these are taken up by the (secondary) yolk sac. Once the placenta is developed, hemo-trophic nutrition with exchange between the maternal and fetal circulation becomes predominant [48]. Even though we have previously shown that maternal diet also affects development and function of the placenta, the specific effect of periconceptional maternal protein intake on placental health fell beyond the scope of our study [49]. Another explanation may be that the relative effect of maternal protein intake is smaller later in pregnancy, because of an increasing role of other (non-nutritional) factors. Finally, less standardized methods of measurement may explain the attenuation. Fetal growth parameters and birthweight were retrieved from medical records and therefore measurement errors cannot be ruled out.

#### 4.2.2. Maternal Animal Protein Intake

The findings of the current study suggest that the positive association of maternal protein intake during the periconception period with prenatal growth and birthweight can be predominantly ascribed to protein intake from animal sources. Periconceptional maternal animal protein intake was statistically significantly associated with increased embryonic growth, and also with higher EFW and birthweight, though not statistically significantly. In line with these results, an additional analysis including an ‘animal/plant protein intake’ ratio (data available on request) showed that consuming more animal proteins relative to plant proteins was associated with increased prenatal growth and birthweight. These findings correspond with other studies that showed a positive association between maternal animal protein intake and fetal growth and birthweight [8,10,11,45]. To our knowledge, the current study is the first study that investigated the effect of protein sources on embryonic growth. In our study population, the CRL and EV of embryos of women with +2SD animal protein intake (absolute increase of 30 g/day) were 0.45 mm (+4.9%) and 0.03 cm^3^ (+34.0%) larger at 7 weeks of pregnancy and 0.97 mm (2.3%) and 0.54 cm^3^ (7.3%) larger at 11 weeks of pregnancy compared to those of women with the mean animal protein intake. As an illustration, the CRL and EV of embryos of women who did not use folic acid supplements or started folic acid supplement use post-conception were 0.75 mm (−7.8%) and 0.01 cm^3^ (−19.5%) smaller at 7 weeks of pregnancy and 1.63 mm (−3.7%) and 0.86 cm^3^ (−12.2%) than those of women who started folic acid supplement use preconceptionally [7,35].

#### 4.2.3. Maternal Plant Protein Intake

Surprisingly, no associations were found between periconceptional plant protein intake and prenatal growth. Previous research on maternal plant protein intake and prenatal growth and birthweight is limited and yielded inconsistent results [8,10,11]. One study found a positive association with birthweight, while other studies found a non-linear (inverse U-curve) association [8,10,11]. Our findings may be partly explained by low digestibility of plant proteins due to anti-nutritional factors and fibers in plant protein sources [2,50]. Moreover, in general, animal proteins are complete protein sources providing all essential amino acids, while plant proteins are deficient in one or more essential amino acids, such as lysine and threonine [2,5]. Therefore, without careful assortment of plant protein sources, the amino acid requirements for fetal growth may not be met [2]. The results may also be related to the differences in micronutrient content. For example, compared to animal protein sources, plant protein sources contain less iron, which is important for fetal growth [5]. However, the effect estimates of the model adjusted for iron intake were comparable to those of the other models, which suggests that iron intake did not affect the relationship between animal protein intake and embryonic growth. Lastly, dietary exposure to pesticides used in agriculture may have affected the associations between periconceptional maternal plant protein intake and prenatal growth. However, further research should be undertaken to investigate this.

### 4.3. Strengths and Limitations

The most important strength of this study is the extensive longitudinal data collection providing information on many patient characteristics and fetal growth. Potential confounding bias is limited as the analyses were adjusted for a comprehensive set of covariates [32,33,34,35,36,37,38,39]. Nevertheless, considering the complexity of the effect of maternal nutrition on prenatal growth, residual confounding from unobserved and unknown factors may still be present. However, as adjusting for covariates had little effect on the effect estimates, we do not think that residual confounding can explain our findings.

Furthermore, the serial 3D ultrasound scans performed in the first trimester of pregnancy by trained medical doctors are unique. Using VR systems and V-Scope software developed at the Erasmus MC accurate CRL and EV measurements were performed, which have been proven reliable (ICC > 0.99) [7,20]. Moreover, in order to assess maternal protein intake, a validated FFQ was used, as it provides semi-quantitative information on macronutrient and energy intake during four weeks within the periconception period [25]. Even though the FFQ is a validated questionnaire, response bias, in particular social-desirability bias, cannot be ruled out. To reduce the number of patients underreporting their dietary intake, we applied the Goldberg cut-off, which was based on the personalized basal metabolic rate and physical activity [27,28]. Additionally, the study population was restricted to patients with a normal BMI, which revealed stronger effect estimates, but no substantial changes of the associations. This indicates limited bias by overreporting or underreporting dietary intake, though underestimation of our results is more likely than overestimation [41,42].

The recruitment of patients in a tertiary hospital may have affected the generalizability of our results. However, the protein intake as percentage of energy intake was 15.2% in our study population, which is between 10% and 20%, as recommended for adults in the general population by the EFSA Panel on Dietetic Products [31]. Even though the patients included in our study are at higher risk of developing pregnancy complications, we think that the direction of the associations is the same as in the general population.

### 4.4. Implications for Future Research

The results of our study suggest that maternal protein intake during the periconception period, and in particular protein intake from animal sources, has a significant effect on embryonic growth and potentially on fetal growth and birthweight. The impact is substantial, since prenatal growth can affect health later in life, and even health of future offspring [6]. Even though knowledge regarding optimal growth of an embryo is still largely unknown, smaller embryos have been associated with fetal growth restriction, reduced birthweight and an increased risk of cardiovascular risk factors later in life [13,14,15,19]. As previous research has demonstrated that maternal diet hardly changes during pregnancy, early health interventions should target women already in the preconception period [51]. These interventions should aim for a well-balanced diet from a variety of sources containing an appropriate intake of macro- and micronutrients. Future research on amino acid composition, nutrient metabolism, digestibility and the interaction with microbiota are warranted to further investigate the association between protein sources and prenatal growth and birthweight. Future studies should also take into account the residues of pesticides and veterinary medicine in plant and animal products, since they have been associated with endocrine disruption and teratogenic effects [52,53,54].

## 5. Conclusions

This study has shown that even in women with a protein intake that met the nutritional recommendations, periconceptional total protein intake and animal protein intake was associated with increased embryonic growth. In addition a positive association was found between periconceptional maternal total and animal protein intake with fetal growth and birthweight, though not statistically significant. This study was, however, unable to demonstrate an association between periconceptional maternal plant protein intake and prenatal growth and birthweight. Our findings highlight the importance of a well-balanced diet during the periconception period and its impact on prenatal growth.

## Figures and Tables

**Table 1:** Baseline characteristics of the study population and stratified by tertiles of total protein intake as percentage of energy intake.

Maternal Characteristics	Total Study Population (*n* = 501)	Tertile 1 (*n* = 167) 8.3–14.1%	Tertile 2 (*n* = 167) 14.2–16.0%	Tertile 3 (*n* = 167) 16.1–23.4%
Age at conception (years)				
Mean (SD)	32.7 (4.3)	32.1 (4.5)	32.8 (4.4)	33.3 (3.9)
Missing	0	0	0	0
Geographical background				
Non-western	50 (10.4%)	21 (13.0%)	14 (8.8%)	15 (9.4%)
Western	431 (89.6%)	141 (87.0%)	145 (91.2%)	145 (90.6%)
Missing	20	5	8	7
Educational level				
Low	33 (6.9%)	20 (12.3%)	9 (5.6%)	4 (2.5%)
Medium	159 (33.0%)	64 (39.5%)	45 (28.1%)	50 (31.3%)
High	290 (60.2%)	78 (48.1%)	106 (66.3%)	106 (66.3%)
Missing	19	5	7	7
Parity				
Nulliparous	205 (42.4%)	64 (39.5%)	69 (42.9%)	72 (45.0%)
Multiparous	278 (57.6%)	98 (60.5%)	92 (57.1%)	88 (55.0%)
Missing	18	5	6	7
Conception mode				
IVF/ICSI	271 (54.1%)	87 (52.1%)	87 (52.1%)	96 (57.5%)
Natural	230 (45.9%)	80 (47.9%)	80 (47.9%)	71 (42.5%)
Missing	0	0	0	0
Body Mass Index (kg/m^2^)				
Mean (SD)	24.7 (4.1)	24.7 (4.2)	24.6 (4.5)	24.6 (3.5)
Missing	0	0	0	0
Folic acid supplement use				
Inadequate	62 (12.9%)	24 (14.8%)	25 (15.5%)	13 (8.2%)
Adequate	420 (87.1%)	138 (85.2%)	136 (84.5%)	146 (91.8%)
Missing	19	5	6	8
Smoking				
Yes	66 (13.7%)	29 (17.9%)	23 (14.3%)	14 (8.8%)
No	417 (86.3%)	133 (82.1%)	138 (85.7%)	146 (91.3%)
Missing	18	5	6	7
Alcohol				
Yes	139 (28.8%)	39 (24.1%)	60 (37.3%)	40 (25.0%)
No	344 (71.2%)	123 (75.9%)	101 (62.7%)	120 (75.0%)
Missing	18	5	6	7
Drugs				
Yes	10 (2.1%)	6 (3.7%)	3 (1.9%)	1 (0.6%)
No	473 (97.9%)	156 (96.3%)	158 (98.1%)	159 (99.4%)
Missing	18	5	6	7
Energy intake (kcal/day)				
Mean (SD)	1940 (562)	2100 (699)	1990 (498)	1740 (383)
Missing	0	0	0	0

Tertile 1 includes the lowest total protein intake (8.3% of energy) and Tertile 3 includes the highest total protein intake (23.4% of energy). Continuous data are presented as means with standard deviation (SD) and categorical data as numbers of individuals with percentages.

**Table 2:** Periconceptional maternal protein intake.

Maternal Nutrient Intake	g/Day	% of Energy	% of Protein
Total protein	72.8 (20.5)	15.2 (2.4)	
Animal protein	41.4 (15.8)	8.6 (2.6)	56.0 (10.6)
Plant protein	31.4 (10.1)	6.5 (1.3)	44.0 (10.6)
RDI first trimester	59.3 (10.5)		
RDI second trimester	67.3 (10.5)		
RDI third trimester	86.3 (10.5)		

Data are presented as means with standard deviation (SD). RDI = Recommended Daily Intake.

**Table 3:** Associations between periconceptional maternal protein intake for each additional 10 g/day and embryonic growth measurements.

Total Study Population	Model 1		Model 2	
(*n* = 501)	β (95% CI)	*p*-Value	β (95% CI)	*p*-Value
√CRL (mm)				
For each additional 10 g/day				
Total protein intake	0.024 (0.002, 0.046)	0.031	0.022 (−0.001, 0.045)	0.061
Animal protein intake	0.025 (0.003, 0.047)	0.027	0.023 (−0.0002, 0.046)	0.052
Plant protein intake	0.011 (−0.031, 0.054)	0.597	0.0001 (−0.045, 0.046)	0.998
∛EV (cm^3^)				
For each additional 10 g/day				
Total protein intake	0.011 (0.001, 0.022)	0.033	0.014 (0.003, 0.025)	0.017
Animal protein intake	0.012 (0.002, 0.023)	0.024	0.015 (0.003, 0.026)	0.012
Plant protein intake	−0.004 (−0.024, 0.016)	0.698	−0.01 (−0.028, 0.016)	0.617

Model 1 is adjusted for GA and energy intake. Model 2 is as Model 1, but additionally adjusted for age, BMI, geographical background, educational level, parity, conception mode, fetal sex, smoking, and folic acid supplement use. In both models animal and plant protein intake were mutually adjusted for each other. BMI = Body Mass Index; CRL = Crown-Rump Length; EV = Embryonic Volume.

**Table 4:** Associations between periconceptional maternal protein intake for each 10% increase as percentage of energy intake and embryonic growth measurements.

Total Study Population	Model 1		Model 2	
(*n* = 501)	β (95% CI)	*p*-Value	β (95% CI)	*p*-Value
√CRL (mm)				
For each 10% increase				
Total protein intake	0.118 (0.015, 0.222)	0.025	0.119 (0.009, 0.229)	0.035
Animal protein intake	0.113 (0.009, 0.216)	0.034	0.011 (0.004, 0.220)	0.049
Plant protein intake	0.083 (−0.127, 0.294)	0.437	0.043 (−0.187, 0.273)	0.716
∛EV (cm^3^)				
For each 10% increase				
Total protein intake	0.048 (−0.0004, 0.097)	0.052	0.061 (0.008, 0.114)	0.024
Animal protein intake	0.050 (0.002, 0.099)	0.043	0.062 (0.009, 0.115)	0.021
Plant protein intake	−0.020 (−0.120, 0.081)	0.701	−0.028 (−0.138, 0.083)	0.623

Model 1 is adjusted for GA and energy intake. Model 2 is as Model 1, but additionally adjusted for age, BMI, geographical background, educational level, parity, conception mode, fetal sex, smoking, and folic acid supplement use. In both models animal and plant protein intake were mutually adjusted for each other. BMI = Body Mass Index; CRL = Crown-Rump Length; EV = Embryonic Volume.

**Table 5:** Associations between periconceptional maternal protein intake for each additional 10 g/day and embryonic growth measurements adjusted for iron intake.

Total Study Population	Model 2		Model 3	
(*n* = 501)	β (95% CI)	*p*-Value	β (95% CI)	*p*-Value
√CRL (mm)				
For each additional 10 g/day				
Total protein intake	0.022 (−0.001, 0.045)	0.061	0.028 (0.003, 0.054)	0.027
Animal protein intake	0.023 (−0.0002, 0.046)	0.052	0.027 (0.001, 0.053)	0.040
Plant protein intake	0.0001 (−0.045, 0.046)	0.998	0.0001 (−0.046, 0.046)	0.998
∛EV (cm^3^)				
For each additional 10 g/day				
Total protein intake	0.014 (0.003, 0.025)	0.017	0.018 (0.006, 0.031)	0.004
Animal protein intake	0.015 (0.003, 0.026)	0.012	0.015 (0.032, 0.026)	0.012
Plant protein intake	−0.01 (−0.028, 0.016)	0.617	−0.006 (−0.028, 0.016)	0.617

Model 2 is adjusted for GA, energy intake, age, BMI, geographical background, educational level, parity, conception mode, fetal sex, smoking, and folic acid supplement use. Model 3 is as Model 2, but additionally adjusted for iron intake. In both models animal and plant protein intake were mutually adjusted for each other. BMI = Body Mass Index; CRL = Crown-Rump Length; EV = Embryonic Volume.

**Table 6:** Associations between periconceptional maternal protein intake for each additional 10 g/day and embryonic growth measurements in women with a normal BMI.

Women with a BMI: 18.5–25 kg/m^2^	Model 2		Model 3	
(*n* = 286)	β (95% CI)	*p*-Value	β (95% CI)	*p*-Value
√CRL (mm)				
For each additional 10 g/day				
Total protein intake	0.046 (0.017, 0.076)	0.002	0.044 (0.012, 0.076)	0.007
Animal protein intake	0.047 (0.017, 0.077)	0.002	0.044 (0.012, 0.075)	0.007
Plant protein intake	0.036 (−0.021, 0.094)	0.212	0.020 (−0.042, 0.082)	0.519
∛EV (cm^3^)				
For each additional 10 g/day				
Total protein intake	0.016 (0.002, 0.031)	0.021	0.017 (0.002, 0.033)	0.024
Animal protein intake	0.016 (0.003, 0.030)	0.021	0.018 (0.003, 0.033)	0.023
Plant protein intake	0.001 (−0.027, 0.029)	0.939	0.001 (−0.030, 0.031)	0.973

Model 2 is adjusted for GA and energy intake. Model 3 is as Model 2, but additionally adjusted for age, BMI, geographical background, educational level, parity, conception mode, fetal sex, smoking, and folic acid supplement use. In both models animal and plant protein intake were mutually adjusted for each other. BMI = Body Mass Index; CRL = Crown-Rump Length; EV = Embryonic Volume.

**Table 7:** Associations between periconceptional maternal protein intake for each additional 10 g/day and standardized EFW and birthweight (*z*-scores).

Total Study Population	Model 1		Model 2	
	β (95% CI)	*p*-Value	β (95% CI)	*p*-Value
Standardized EFW (*n* = 422)				
For each additional 10 g/day				
Total protein intake	0.062 (−0.025, 0.150)	0.161	0.060 (−0.029, 0.150)	0.186
Animal protein intake	0.064 (−0.024, 0.151)	0.155	0.062 (−0.028, 0.151)	0.178
Plant protein intake	0.032 (−0.131, 0.194)	0.702	0.021 (−0.151, 0.193)	0.810
Birthweight (*n* = 501)				
For each additional 10 g/day				
Total protein intake	0.049 (−0.036, 0.134)	0.259	0.019 (−0.069, 0.107)	0.667
Animal protein intake	0.049 (−0.036, 0.134)	0.256	0.019 (−0.069, 0.107)	0.675
Plant protein intake	0.038 (−0.123, 0.198)	0.647	−0.001 (−0.174, 0.173)	0.993

Model 1 is adjusted for energy intake. Model 2 is as Model 1, but additionally adjusted for age, BMI, geographical background, educational level, parity, conception mode, fetal sex, smoking, and folic acid supplement use. Fetal sex was not included in the models for standardized birthweight. BMI = Body Mass Index; EFW = Estimated Fetal Weight.

## Data Availability

The data presented in this study are available on request from the corresponding author.

## Supplementary Materials

The following supporting information can be downloaded at: https://www.mdpi.com/article/10.3390/nu14245309/s1, Table S1: Baseline characteristics of the included and excluded pregnancies.
