# Supplementary material for: Periconceptional Maternal Protein Intake from Animal and Plant Sources and the Impact on Early and Late Prenatal Growth and Birthweight: The Rotterdam Periconceptional Cohort
Source: Nutrients. 2022 Dec 14;14(24):5309. doi: 10.3390/nu14245309 (PMC9785913; doi:10.3390/nu14245309)
Supplement: Supplementary file 1 [file nutrients-14-05309-s001.zip › nutrients-2057068-supplementary.pdf]

Table S1. Baseline characteristics of the included and excluded pregnancies.

Maternal characteristics	Total study population (*n* = 501)	Excluded pregnancies (*n* = 864)
Age at conception (years)		
Mean (SD)	32.7 (4.3)	32.0 (4.8)
Missing	0	158
Geographical background		
Non-western	50 (10.4%)	104 (16.6%)
Western	431 (89.6%)	523 (83.4%)
Missing	20	237
Educational level		
Low	33 (6.9%)	50 (8.0%)
Medium	159 (33.0%)	225 (35.9%)
High	290 (60.2%)	352 (56.1%)
Missing	19	237
Parity		
Nulliparous	205 (42.4%)	238 (38.0%)
Multiparous	278 (57.6%)	389 (62.0%)
Missing	18	237
Conception mode		
IVF/ICSI	271 (54.1%)	166 (35.7%)
Natural	230 (45.9%)	299 (64.3%)
Missing	0	399
Body Mass Index (kg/m^2)		
Mean (SD)	24.7 (4.1)	27.1 (5.5)
Missing	0	265
Folic acid supplement use		
Inadequate	62 (12.9%)	154 (24.7%)
Adequate	420 (87.1%)	469 (75.3%)
Missing	19	241
Smoking		
Yes	66 (13.7%)	90 (14.4%)
No	417 (86.3%)	534 (85.6%)
Missing	18	240
Alcohol		
Yes	139 (28.8%)	184 (29.5%)
No	344 (71.2%)	440 (70.5%)
Missing	18	240
Drugs		
Yes	10 (2.1%)	13 (2.1%)
No	473 (97.9%)	611 (97.9%)
Missing	18	240
Energy intake (kcal/day)		
Mean (SD)	1940 (562)	1590 (678)
Missing	0	386

Continuous data are presented as means with standard deviation (SD) and categorical data as numbers of individuals with percentages.
